# Practical Essays on Medical Education and the Medical Profession

**Published:** 1829

**Authors:** 


WESTERN JOURNAL

OF THE

MEDICAL & PHYSICAL SCIENCES.

OCTOBER, NOVEMBER, & DECEMBER, 1829.

### ESSAYS AND CASES.

Art. I.—*Practical Essays on Medical Education and the Medical Profession, in the United States.*—By The Editor.

### ESSAY II.

### Private Pupilage.

It is impossible to approach this subject without emotion. I know of no other, the free discussion of which would be so likely to agitate the profession in America. That I shall treat it without prepossession, prejudice or passion, is not probable; nor is it certain, that I shall say all which should be said; but intending to write with courage and condour, I may hope to awaken, if not to guide, the spirit of reform.

To come at once, to a full understanding with the reader, I shall assume the general proposition, that *our system of private tuition is essentially bad.* But, in truth, we have *no system;* and it would be more correct to say, that in the United States *a good system of private pupilage is imperiously required.*

In the preceding essay, on the preparatory education of students, I have endeavored to show, that its errors and imperfections are permanently stamped on our *literary* character. If this be the fact, we can scarcely doubt, that a defective elementary education, in medicine, must greatly retard our advancement to *professional* excellence; and whatever does this, demands the gravest attention.

The ordinary time of private instruction is so short, as to require that it should be well employed. If the rudiments of the profession are not *then* acquired, they are seldom properly understood. It is not easy, afterwards, to supply the omission, or correct the errors of that period. I have never known it done. Every stage of life has its peculiar tastes and appropriate studies. In youth we acquire elementary truths—in manhood arrange them into general principles—in the meridian of life, apply them to the production of practical results. Such is the law of nature; and was it inscribed on the doors of every medical library, they would not be so often opened to no beneficial effect.

Having spoken at large of the selection of pupils, I come now to the choice of preceptors. This is a point of more importance than most parents suppose. Many of them, indeed, seem to act on this important subject with but little discrimination. The circumspection with which they select masters, when about to apprentice their sons to mechanical occupations, is seldom manifested when a medical education is the object. They appear indeed to feel themselves incompetent to judge, in the latter case, and, generally, consult economy or convenience; although in so doing, they not unfrequently determine for their sons, a far more imperfect and limited destiny than they intend.

1. It is not *necessary* that the preceptor should be a man of genius; but it is indispensable that he should possess a sound and discriminating judgment, otherwise he will be a blind guide. Of his qualifications in this respect, every parent with opportunities of personal intercourse, may form a correct opinion. It is not requisite, that he himself should be acquainted with the principles of the profession to judge of the talents and common sense of its practitioners: The most unlearned can distinguish between a clouded and an unclouded intellect.

2. A preceptor should be learned, at least in his profession. If a father wishes to make his son a skilful mechanic, he places him with a good workman—not a botch. How can a man direct the studies of a youth through the elements of several different sciences, if his own acquaintance with them is imperfect and confused? As well might architecture be taught by one, who ignorantly combined in the same column, the parts and proportions which belong to all the different orders. To judge of the *attainments* of professional men, is a more difficult task, than to estimate their *abilities;* but although a father may not be able to do this by direct inquiries, he still has much in his power in this respect. The previous opportunities of the individual whom he scrutinizes, taken in connexion with his existing habits, will generally enable him to come to a correct conclusion. If the former have been limited and the latter are idle, he may safely conclude, that the requisite attainments are wanting; and look elsewhere for the aid which is indispensable to a rapid and logical prosecution of studies.

3. It is not sufficient that a private preceptor has talents and learning. He must be devoted to his profession, jealous of its character, and ambitious of its honours. With such feelings, he will awaken high aspirations in the bosom of the youth whose destiny is committed to his keeping, enamour him with the sciences whose rudiments he is to acquire, and animate him in the toil which their difficulties impose.

4. The preceptor should be conscientious in the performance of his duties; that is, he should feel the responsibilities of his office, and studiously endeavour to discharge them. It is easy to deceive a father in regard to the progress of a son, in the study of a profession with which the former is unacquainted, for all his partialities coincide with the flattering report of the master, and cause them to be received with credulity. In this way many a tutor has repressed paternal anxiety, and screened, from paternal vigilance, his inattention and neglect; inspiring high hopes, at the very moment when his own criminal derelictions of duty were sowing the seeds of their future destruction.

5. The private preceptor should, if possible, be a man of business:—Punctual to his pecuniary engagements, accurate in his accounts, and systematic in all his affairs. He will thus be an example for the imitation of his pupils, on points in which too many students of medicine grow up with deplorable and enduring imperfections.

6. Finally, sound morals and chastened habits are not among the least of those qualifications, which an anxious father would require in the man, whose deportment and precepts are to exert so great an influence on the character of his son. It would be superfluous to enumerate all the vices, which ought to disqualify a physician for the private tuition of boys and young men; but there are a few which from their frequency and effect, deserve, on every suitable occasion, to be held up to the scorn of society. A want of attention to professional promises, with a consequent fabrication of excuses and apologies, is a failure which no preceptor can habitually display, with impunity to the morals of his pupil; and ought to be regarded as a disqualification, with whatever genius and learning it might happen to be associated. Gambling is another vice, the morbid influence of which on the plastic and imitative pupil, should be still more seriously deprecated; since in addition to the corruption which it carries into the youthful heart, it diverts from study, generates neglect of business, and leads to the loss of character and fortune. Lastly, intemperance not less than gaming, should displace a physician from the ranks of those whom we entrust with the tuition of young men. Intemperance, in the United States at least, has been regarded as a prevailing vice of the profession, and it has too often happened, that boys almost from necessity have been apprenticed to drunken doctors. I am proud to say, that this necessity is rapidly diminishing. Nevertheless, the number of intemperate physicians is still so great, that parents should proceed with care and caution in the choice of preceptors. One of the greatest calamities they could inflict on a son, would be to place him under the guardianship of a drunkard: if unsusceptible of being corrupted by example, he would suffer numerous embarrassments and mortifications—if susceptible, he must be ruined.

It would not be easy to estimate the injury which has resulted to individuals, to the profession, and to society at large, from a want of discrimination in parents, when about to place their sons to the study of medicine. Many a youth has, in this manner, been sacrificed by an ignorant or thoughtless father. Irresolution has sunk into moral cowardice, dullness declined into stupidity, and genius substituted its flights and phantasies for patient analysis; ambition has found its goal in the acquisition of premature and badly earned certificates and diplomas; idleness relieved itself by ceaseless changes from frivolity to frivolity; and the love of pleasure been left to luxuriate into licentiousness and vice. It is playing at cress purposes, for a father to dispense with the services of his son and incur the expense of his professional education; and, at the same time, to place him under an unqualified or unprincipled master. It is due to themselves and their children, that parents should consider this subject seriously. It is, also, due to the profession and the community, that they discriminate on this point. When they confide their sons to the ignorant, the indolent, or the vicious, they encourage that, which every member of the commonwealth is bound to discountenance; and withhold from the learned and meritorious members of the profession, one of the legitimate rewards of their industry, zeal and perseverance. Could no physician hope to attain the distinction of being a preceptor, without first establishing a character for sound science and good morals, not a few who are now reckless of both, would be held firmly to their acquisition.

Another mistake which requires correction, relates to the time of life when medical studies are commenced. Essentially latitudinarian, the American people allow themselves in this respect, as in many others, a singular degree of liberty. Many boys are put to the study of physic at fifteen, while men of twenty five or upwards, who have been too indolent or too unskilful to succeed in their first pursuits, are every day seen to betake themselves to this most difficult and elevated vocation without a single misgiving. Examples of success might be cited in support of both periods, but still they are extremes. When a boy begins the study at fifteen or sixteen, he generally sets up for himself at nineteen or twenty; a time of life when he lacks *judgment*, whatever may be his acuteness of parts or the extent of his information. On the other hand, he who takes his first lessons after his mind has attained maturity, will seldom move with ease in the practical duties of the profession, even if his life have been spent in studies up to that period; and, if this should not have been the case, which in America is the general fact, the chances are altogether against his ever becoming useful or acquiring public confidence. Seventeen or eighteen, seems to me the most proper age to commence the study. By that time, a due preparatory education may be acquired; the mind and *hands* are still so plastic, as to be directed in their operations; the reasoning faculties are sufficiently developed to investigate the principles of the profession; and the period between that and twenty one or twenty two, is about long enough to admit of the necessary acquisitions.

The youth of the United States, are not only put to the study of medicine at improper ages, but the period of their studies is too short. Nothing is more common than for them to enter on the practice, at the end of two years, or even eighteen months, and three years are thought to be a protracted and tiresome pupilage. But I do not hesitate to assert, that even that term is too short; and that four years should be considered as indispensable. Of the various causes, which have retarded the advancement of the profession in this country, and inflicted upon it such multitudes of medical practitioners who leave behind them no single monument of skill or science, this is one of the most operative and universal.— The blame rests, in part, on our *national* impatience to engage in practical exertions, but still more on the custom which prevails among fathers who are indigent or but little above that condition, of devoting their sons to the profession. The term of their pupilage, is thus determined, not by the sciences which they ought to study, but their means of support.

The situation and circumstances in which a pupil should prosecute his studies, deserve to be considered. In the country and all the smaller towns of the United States, the fashion is for the student to reside and study in the *shop* or office of his preceptor, and often to become a member of his family.—The latter has its advantages, as contributing to preserve him from dissipation; but his time is too often wasted in labours foreign to his studies, and he is apt to be introduced more frequently into company, than is compatible with his interests as a student. Of the *office*, as a place for study, "much might be said on both sides." It is the resort of many persons besides those who call for medical assistance, and the student is subjected to perpetual, if not protracted interruptions. In this way he loses many precious hours, which can never be reclaimed. At first, the effect of these interruptions is so distracting, as materially to impede his progress; but their influence diminishes with the repetition, until at last he comes to form habits that are not without their value in after life. To understand this, we need only recollect, that the practice of his profession will subject him to incessant interruptions of a similar kind; and if he has not early acquired the art of reading in the midst of them, he will not read at all. From the want of this habit, many physicians pass their lives incapable, as they conceive, from the vexations of business, of reading as many volumes, as they should study every year. Another compensating benefit, is the practical facility which they acquire, from witnessing and assisting in the various little clinical and surgical operations of which a Doctor's shop is the theatre. This however, is not without its disadvantages, as the student is liable to have his mind bent upon practical matters, long before he is able to comprehend their *rationale;* by which his attention is diverted from elementary studies, and a foundation laid for future empyricism. Of the different modes of generating quacks, this is the most prolific; and, as they bear the external marks of legitimacy, the most pernicious to society. Every student, however, should spend a part of his pupilage among the *officinal* substances, that are the agents with which future objects are to be accomplished. He should learn their sensible qualities by observation, and become familiar with all the compounds and pharmaceutic processes of the shop. But to acquire this knowledge, it is only necessary that he should be in the office, or in the shop of an apothecary, while prosecuting the study of chemistry and pharmacy. When engaged in anatomy, physiology, pathology and botany, the dissecting room, his chamber and the fields, are his appropriate situations. After having, however, acquired a competent knowledge of those branches, with the principles of surgery and the classification of diseases, he may and should return to the scenes of practical medicine; for he is then prepared to comprehend the nature of what he sees, and, to a certain extent, can engage understandingly in the duties of the profession. If, at this period, he be kept in retirement, indecision of character may be generated, and his future usefulness materially abridged.

The portion of each day, which pupils in medicine may devote to their studies, consistently with good health and the future welfare of their constitutions, must necessarily vary in different persons; but a safe average would be one half, or twelve hours. This would give seven for sleep—and few young persons can do with less—two for meals, and three for exercise, labour and society. The last of these divisions is abridged by some and lengthened by others; but in general is profitless to all. With most pupils, the hours of relaxation from study, are hours of idleness, pleasure and gossip: Too often of downright listlessness, or ruinous dissipation. But whether whiled or rioted away, they bring no renovation either of mind or body. To answer the end for which they are set apart, they must be spent in active exertion in the open air; which will not only prepare the mind for new labours, but ward off dyspepsia, palpitations, hypochondraism, and red eyes; and prevent that debility of frame, so falsely regarded as the necessary effect of hard study, when it results from the want of a sufficient amount of hard labour.

Is it necessary or advisable, that the student of medicine should prosecute his studies on Sunday? In answering this question, I shall of course speak not as a divine, but a physician and teacher. I would say, then, that it is not, but the reverse. As a general rule, his progress will be greater, if he suspend, than continue his studies through the sabbath. The mind, no less than the body, requires not mere moments of relaxation, but hours of actual repose, at least from the particular labours in which it is engaged. Moreover, the student comes into his medical pupilage, with established habits in this respect, which cannot be violated with impunity.— Finally, the moral and devotional feelings require cultivation, and this can best be done by the recurrence, at stated times, of a day for that special object. On the whole, therefore, I regard those preceptors, who encourage their pupils in the prosecution of medical studies on the sabbath, as committing an error in their plan of professional education.

Closely connected with this subject, is another of deep interest to parents as well as pupils. Few things exert a greater influence on our prosperity, than a systematic attention to pecuniary accounts, and the observance of a judicious economy in our expenditures; all of which must be practised in youth, to become matters of habit in manhood. When a father sends his son from home to study the profession, he should decide upon the style of living (always simple and unostentatious) in which the young man is to pass his apprenticeship; and, furnishing him, from time to time, with the necessary means, should require of him to render at stated periods an account of their appropriation. This would compel him not only to limit his expenditures to his resources, but give him at an early period, a habit of recording his disbursements and keeping his accounts, that would be of great advantage in all after years. I have known many physicians, who were neither accurate accountants nor good economists, chiefly because they had grown up in the habitual neglect of the duties which I have indicated.

Other studies than medicine, should occupy a portion of the students time. In the preceding essay, I have said, that a majority of our students of medicine, particularly those of the West, enter upon their pupilage with a most incompetent education. For all such there is no alternative, but to cultivate the elements of literature and science during their medical pupilage, or to remain superficial scholars throughout their lives. I regret to say, that the majority choose the latter. But are those who have enjoyed the advantages of a collegiate education, exempted from the necessity which rests upon their less favoured brethren? It is obvious that they are not. Few minds are so retentive as to keep what they have acquired at school, if they do not add to it, by a subsequent cultivation of the same branches. To secure this important object, it is absolutely necessary, that the student should devote to it a stated portion of every day—for example the morning or evening; and suffer no controllable circumstance to interfere with his plan. A small part of his time thus occupied, will in the course of four or five years make him a passable scholar, should his learning have been even at the *minimum;* but when he has the happiness to engage in professional studies with the attainments of a *bachelor*, he may by the course here recommended, so far extend his literary acquirements, as to enter on the theatre of life with an enviable character for sound erudition.

It ought to be mortifying to our national pride, to observe how few of those who might thus become ripe scholars ever attain to that elevation. Even many of our writers, except by the latin mottos and quotations with which they seek to adorn their writings, would not be suspected of having pretensions to a liberal education; while to the majority of their professional readers, the whole garniture of classical common places, is occult and unintelligible.

The practical branches of literature and science, on which the student of medicine should devote a portion of his time are:—

First, English grammar and the art of composition; without an attention to which, *after* the commencement of his medical studies, no young man need hope to become even a tolerable writer.

Secondly, Physical geography, embracing the leading facts in meteorology, which constitutes a foundation for the study of the physical condition and diseases of man, in the various countries and climates of the earth; a comprehensive view which should be taken by every physician.

Thirdly, The outlines of history; that he may be able to trace the progress of the profession through the different nations which have transmitted it to his own; and come to understand the influence of moral causes upon the physical system, whose aberrations he is to rectify.

Fourthly, The elements of mathematicks and natural philosophy; not merely for the purpose of invigorating his reasoning powers, but because the latter has many points of illustrative association with medicine.

Fifthly, The French language; since the subjects of morbid anatomy, physiology and pathology, have been recently cultivated with extraordinary success by the French physicians; but a small part of whose voluminous and classical writings, are translated into our vernacular tongue. To avail himself of this source of improvement, it is not necessary that the student should conquer the difficulties of the French pronunciation. A few elementary books, with or without a teacher, are all that are requisite to open to him the rich treasures of medical knowledge, which that enterprising people have accumulated.

Sixthly, The Latin and Greek languages. Of all the branches of literature which have been named, it is, perhaps, most necessary, for those who would retain or establish the character of good scholars, to continue the study of these languages, through the period of their medical pupilage.—Their daily readings will extend their acquaintance with our own language; but without a special effort their classical knowledge will speedily depart from them. But few students are apprized of this fact, until they learn it from experience; when the discovery in general comes too late to be useful; for that which is lost by indolence is seldom reclaimed by industry. A few minutes of daily attention to Greek and Latin, would not only prevent deterioration, but ensure actual advancement; while a longer, but not objectionable period, would bring an uneducated pupil, to such an acquaintance with the grammar and vocabulary of those languages, as would prove in the end quite equal, to all that is generally left at forty, of the classical acquisitions of twenty.

Having disposed of the chief preliminary topics, we are prepared for inquiries of greater technicality and moment; and shall next consider the plan on which the pupil should be conducted through his professional studies. 'Method,' says Linnæus, 'is the soul of science,' an apothegm to which most private preceptors in this country seem to be strangers. With their own professional knowledge unarranged, but provided with a small assortment of medicines, a meagre collection of books, chiefly, practical compilation and compends, a few surgical instruments, and the fragments of a skeleton, they set up for preceptors; as unconscious of their incapacity, as they are ignorant of the defects in their means of instruction.—Their case, however, is far from hopeless, for in fact they win the confidence of the publick by the palpable honesty, with which they set forth their pretensions to its patronage. A student once acquired, he is turned loose in the *shop*, and, left to cater for himself, at once becomes a real *eclectic*. Generally, however, he begins with descriptive anatomy; which, proving a *dry* pursuit, he abandons in due season, and seldom fails to hit upon materia medica, from which he proceeds to the practice of physic, obstetrics and surgery. He is now on elevated ground, and looks down on chemistry, pharmacy, botany, general anatomy, physiology and pathology, as matters well enough in their place, but lying so far below, that he cannot descend to them without disgrace. This occupies the first year; when being *qualified* to 'practise,' or at least for 'seeing practice,' his mind turns upon recepies; and he spends the second year, in collecting and cramming his master's patients with 'infallible cures.' After this, according to his pecuniary means, he either attends lectures, or 'opens shop' for himself. For the first half of this *course of studies*, the preceptor generally does not think it worth while to interfere; but the second passes under his watchful eye, and he is thus enabled to certify, that his *eleve* is amply prepared for the duties of the profession, and promises fair to become an ornament of the profession!

In medicine, as the other sciences, that method is best, which requires the student to take nothing on trust—to anticipate no principle or leading fact. The whole course should, as far as possible, be purely synthetical.

In conformity with this opinion, he ought to begin with CHEMISTRY; provided that science had not made a part of his academical or collegiate studies. It is necessary, however, that he should go extensively or minutely into the practical details of this science; as the subject is to prepare him for a proper understanding of the chemical terms, with which he will meet in the study of physiology.

Relinquishing chemistry he should engage in special ANATOMY, which may be studied in the following order;—1—the bones, 2—the muscles, 3—the viscera, 4—the blood and absorbent vessels, 5—the brain and nerves. He is now prepared for general anatomy, and should make himself familiar with the anatomical relations and dependencies of the various organs.

He will thus qualify himself for the study of PHYSIOLOGY, the foundations of which are anatomy and chemistry; and his progress, in that important branch of the profession, will, *cæteris paribus*, be in proportion to the accuracy of his anatomical and chemical knowledge.

From physiology he may either recur to the studies of which chemistry is the basis; or, according to his taste or the judgment of his teacher, proceed by a natural transition to acquire an outline of PATHOLOGY. The latter is perhaps, on the whole, the better course; for when the mind of a pupil is deeply interested in the contemplation of the healthy functions, it passes with facility to the study of their disordered states; and thus, with little difficulty, comes to comprehend the general principles of pathology: But he should, on no account, be permitted to stray beyond the rudiments of the science of diseased actions. And here I will take occasion to remark, that I know of no distinct treatise on the elements of pathology which is adapted to this stage of a pupil's studies; and, hence, it is necessary, that a selection should be made, by the preceptor, of such parts of various works on the theory and practice of medicine and surgery, as will convey a correct general idea of the existing state of pathological science.

Having gone thus far in the study of the animal economy, in its healthy and morbid conditions, he must stop. In going further, he would instantly reach matters which he could not understand, for want of an acquaintance with branches which I shall now proceed to enumerate.

The first of these is Botany. It requires but little observation to perceive, that the science of the vegetable kingdom has many important relations with medicine. Plants and animals offer numerous anologies, in their structure, and the physiology of one, contributes to enlighten us, in reference to the other. But the connexions of botony with materia medica, are still more intimate than with physiology, and cannot be overlooked by the most unobserving; and yet the majority of physicians are as ignorant of that beautiful science, as of algebra or astronomy. It is by no means necessary, that a physician should be a *practical* botanist; but it argues little for the taste or ambition of a student of medicine, that he is content to remain ignorant of the fundamental laws of any department of organized nature. The fashionable excuse for making a *pons asinorum* of the science of botany, is the extent and difficulty of its nomenclature. But admitting, that in this respect, it even transcends anatomy, while its utility is comparatively limited; I can perceive nothing in the case which should frighten a young man of courage and inquisitiveness, from the undertaking. If his knowledge of the Greek and latin languages is considerable, the technicology of botany, can present no obstacles which a little resolute industry will not remove; and if he is nearly ignorant of those languages, the condition of most medical students, the study of botanical terms, will materially improve his knowledge of their vocabularies. In botany, more than most other sciences, that which is repulsive lies at the entrance. It might be likened to a garden hedged in with thorns and thistles: the tangled enclosure once penetrated, a rich possession of flowers and fruit is the reward. In addition to its utility, as a collateral aid to physiology, and an introduction to materia medica, there is another advantage in the study of botany, which deserves to be set forth. Plants are arranged by their external characters; among which there are innumerable affinities that cannot be seen and contemplated, without imparting acuteness to the faculties of observation, and establishing a habit of natural classification which is beneficial throughout life. In these respects, I know of no science that can be compared with botany.

Having added to his outline of chemistry, a competent knowledge of the first principles of botany, the student approaches the composite science of MATERIA MEDICA, with conscious preparation, and the confidence necessary to success. It is the duty of his teacher to acquaint him with the important fact, that the natural and pharmaceutic history of medicines, is totally distinct from the study of their effects upon the living body, either in health or disease, and should first engage his attention. This, however, is seldom done, and the student is allowed to fix his mind upon the latter, before he has made acquaintance with the former;—an inversion in the order of study, which lays the foundation of many future imperfections. He should, therefore, be held rigorously to the natural history, composition, and preparations, of the different medicines; until he becomes familiar with the whole. He may then proceed to examine into their effects, on the animal system; and here, again, he will be in danger of inverting the order of inquiry. Most students are prone to connect the names of particular medicines with particular diseases, by which they lose the disposition to generalize, and establish wrong associations that often continue throughout life. The first object should be to arrange the articles of the materia medica, according to their effects, not in the cure of diseases, but on the different functions; and beyond this important and difficult task, the pupil at this stage of his studies should never be allowed to proceed; as the very next step would be taken in the dark. It is necessary, therefore, that he should again change his course, and direct his inquiries upon other objects which I shall now proceed to consider.

I will not say, that in returning to the study of diseases, the pupil should review his anatomy, for in fact this fundamental branch of the profession, should not have been neglected in any period of his course, as no kind of knowledge is so difficult to retain. Hence he should, as far as practicable, associate it with all his other knowledge, a measure that will contribute greatly to its retention and usefulness. He should, however, review his physiology; not by re-perusing the authors already gone over, but some treatise or compilation which he had not before read, and which presenting new facts and speculations, or old ones in a new aspect, may clear up his doubts and fix his principles. This being accomplished, he resumes the study of pathology with acquirements that will enable him to discuss the theories of physic and SURGERY to their utmost limits. These theories he is not to regard as separable into distinct sciences, for they are in fact one and indivisible. Their primary elements are the same. Every physician need not be an *operative* surgeon, but every good surgeon must be likewise a physician. The mere operator, if any such there be, is but a mechanic. The amputation of a limb is a mechanical process; but the treatment which might have been employed to subdue inflammation and ward off the gangrene that rendered the operation necessary, could only be guided by the principles of clinical medicine. Take another example. The extraction of the calculus is effected by the use of instruments, moved according to certain *formulæ;* but the diathesis which generates the stone, is among the most occult and complicated subjects which can tax the genius of the physiological pathologist, or animate his clinical efforts. The *desideratum* is, in fact, a method of treatment that will render a resort to the cruel and hazardous operation of lithotomy unnecessary. The surgery of this case, then, as of most others, grows out of the imperfection and constitutes the opprobrium, of its therapeuticks; and the man who might limit himself to the former, would justly be regarded as an empyrick. Again. The whole subject of inflammation, belongs equally to medicine and surgery, and may be studied without a special reference to either, while they contribute alike to the illustration of its origin, progress and termination.

Whatever, then, may be the ultimate aims of different pupils, their pathological studies should be the same. They must seek to acquire distinct and vivid conceptions of the morbid states which are supposed to be indicated by the terms congestion, irritation, inflammation, fever, nervous irritation, morbid sensibility, sympathy, spasm, morbid secretion, metastasis, vicarious action, collapse, torpor, debility, exhaustion, proximate cause, organic læsion, vis medicatrix, obstruction, local determination, malignity, and many others of subordinate moment, which constitute the elements of pathology, whether clinical or surgical. When they have grappled for a sufficient time, with the difficulties which every attempt to learn the true import of these common, but equivocal words unavoidably imposes, they may proceed to consider the *varieties* of inflammation, fever, spasm, and the other morbid states, which will bring them to NOSOLOGY, or the classification of diseases; when for the first time, they will perceive the paths by which those who intend to limit themselves to clinical medicine, and those who propose to cultivate operative surgery, are to diverge from each other. Having looked into the systems of nosology, with sufficient care to perceive, both their importance and their imperfections, the coadjutors may betake themselves in *some* degree to separate studies:—the student of physick to functional, the student of surgery to organic derangements. The latter are, however, in all cases—mechanical injuries excepted—the offspring, as in turn they become the occasion, of the former; and hence they must both be studied by each class of pupils, though not in an equal degree. In his nosological inquiries, the student becomes acquainted with what the naturalists call *essential characters;* but, arriving at the investigation of particular maladies, whether medical or surgical, he will of course extend and correct his knowledge of SYMPTOMATOLOGY; endeavouring by the utmost effort of his understanding to connect each symptoms with the morbid state of which it is the sign. In doing this he will be led to enlarge his knowledge of MORBID ANATOMY, the first ideas of which might have been received while engaged on general pathology.

In this stage of his progress, he is qualified to investigate the causes of disease, a subject so enveloped in obscurity, that many students give it but a passing attention. Aetiology is, however, a branch of medical science of sufficient importance to justify, I should rather say, demand an attentive investigation; and the very difficulties with which it is beset, should stimulate a pupil to its deep and protracted examination.—Scarcely any portion of his studies will bring into requisition all his knowledge of nature and art, like that of which I am speaking. Of the origin, combination and mode of action of the *causas morborum*, much remains undeveloped; partly from the intrinsic difficulty of the subject, but still more from the loose and limited manner in which it has been generally studied. With these convictions, I would indicate it to students of medicine, as a department of the profession in which they may labour with a well founded hope, of doing good to society and credit to themselves.

Finally, he arrives, by a regular transition, at the Practice of the profession—its therapeuticks and operations. He studies the indications of cure, and labours to establish in his mind, correct associations between symptoms and remedies—an association not arbitrary and empyrical, but founded on an ample and accurate knowledge of the functions of the living body, in their regular and irregular conditions; and of the influence of external agents in the production and cure of diseases. The popular distinction between physic and surgery now becomes more apparent than before; and I shall proceed to speak of them separately.

The student who intends to limit himself to the practice of physic, must recollect, that even in countries where the line of separation is drawn with an absurd and unnatural precision, the physician is compelled to prescribe for a great number, and variety of cases which belong equally to the province of the surgeon. If this be the case in Europe, it must be much more so in the United States, where out of the large cities, there are no professed surgeons. In fact, all our physicians are surgeons likewise; and differ from the practitioners so called, only in declining the performance of a few of the greater operations—such as those for hernia, lithotomy, aneurism, deep seated tumours, cataract, and a few others. To the reduction of dislocation and fractures, the management of ulcers, trephining, amputating, dressing wounds, and other duties of a similar kind, which, from their frequency, make up the mass of surgical business, every physician is presumed to be competent. The pupil then, even when engaged in his *practical* studies, is by no means to limit himself to that which is technically called physic; but, extending his inquiries to surgery, stops short of nothing but its higher operations.

Every part of a course of medical studies abounds in difficulties, and calls for intense and sustained application; but no stage is so trying to the powers of the student, as that which may be called the *therapeutick*. Hitherto he has occupied himself, successively, upon distinct sciences, which he perceived to abound in connexions favourable to their union into a system of professional knowledge; and that union, in reference to his own mind, he is now to effect. He is faithfully represented by the commander, who having embodied and equipped a great variety of separate military *corps*, has at length to consolidate them into an army and direct its active operations.

It is difficult to furnish a student with rules for the organization of his various attainments into a practical system, and much must necessarily be left to his own genius and judgment. A few hints, however, may not be without their utility.

1. If he now finds deficiences in any of his preliminary acquirements, he should supply them, without delay, by a recurrence to the branches in which they are discovered to exist.

2. In ascertaining general principles, he should carefully note those which are of a doubtful character, and rest upon them as few rules of practice as possible.

3. His practical maxims, in all cases, should be logical deductions from his principles.

4. If they do not conform to those of the great and original writers of the profession, he should *doubt* their correctness, and act upon them cautiously; but not reject them without a trial.

5. He should recollect that the same diseases, in different countries, frequently require variations in their treatment, and that he must not implicitly adopt the rules of practice that have been found successful elsewhere.

6. When he meets, in practical works, with different modes of treatment for the same disease, he should not suppose that one only can be correct, and the others necessarily erroneous; for diseases may be cured by various methods. In becoming an eclectic, in these cases, he must carefully examine the whole, and test their merits by the great principles of pathology and therapeuticks, which compose his own system. When he attempts to select from among them, he should avoid uniting rules or recipies that are incompatible, and would therefore countervail or neutralize each other.

7. In his practical researches, he should always prefer original works to compilations, and monographs to systems.

8. He must be on his guard against the delusion of a fancied simplicity, in the system which he constructs. Every complex machine is liable to a variety of irregular movements, which can only be reduced to order by a corresponding diversity of means. But of all machines the human body is the most complicated, exhibits the greatest number of disordered actions, all differing from each other, and requires the greatest variety of remedial applications.

9. When arrived at this stage of his studies, he should no longer stand aloof from the practical duties of the profession; but avail himself of frequent opportunities to make an application of his knowledge. This is the end for which he has studied, and his final success will be proportionate to the facility and effect with which he can make such application.—Skill in practice does not arise, however, from the number of cases he may see or treat, so much as the manner in which he contemplates them. Each one should be a study, and all things relating to it, should be connected with the principles that guide him, and which, in turn, they may serve to illustrate or overthrow.

Should the student intend to make Operative Surgery, his principal object, he may observe most of the foregoing suggestions, while he bestows especial attention on the following subjects:—

1. The anatomy of the parts which are the seats of the greater operations of the art. With these parts, particularly such of them as cannot be cut into without danger, he should be perfectly familiar:—Not merely able to enumerate the muscles, and fasciæ, and nerves, and bloodvessels of which they are composed, but to conceive accurately of the relative situation of those anatomical elements. He should, moreover, learn to know each part, not only by the eye, but by the finger; as it will frequently happen, in deep and bloody operations, that the sense of touch is the only one he can employ.

2. He should practise the various operations on the dead subject; for by practice only, can he become adroit, or acquire that confidence which gives self possession in moments of difficulty and doubt. In this stage of his *practical* studies, he will often find it advantageous to supply the deficiency of human subjects, by a resort to dead animals; in the selection of which he will be materially aided by some acquaintance with zoology.

3. He should practise on the living or the dead subject, the application of the various kinds of bandages and apparatus of surgery. For the neat and efficient discharge of this duty, more experience is necessary, than many persons suppose until they come to the trial in cases of real injury, when it is too late to prepare themselves.

I shall conclude this branch of the subject with a reference to OBSTETRICS. This department of the profession is emphatically a compound of physic and surgery, and on this account not less than many others, it should be the last which a pupil studies. The healthy functions of the uterine system however, will have been leared by him, as a portion of physiology. As to obstetrical studies, the most important observation I can make, is, that the young physician will be called to act, with less of *practical* information, than in any other branch of the profession, for in no stage of his pupilage can he have many opportunities of acquiring that kind of knowledge. Hence he should study the subject in books, and by means of plates and models, with extraordinary care and diligence.—Without doing this, he may be thrown into situations of responsibility, most harrowing to his feelings, if not fatal to his patient.

With these intimations, I shall close what it seems necessary to say, concerning the plan on which a course of medical surgical and obstetrical studies should be conducted; and in conclusion, shall speak of the aid which the pupil should receive from his preceptor.

At the risk of being thought severe, and perhaps tedious, I feel constrained to repeat, as a general fact, that the physicians of the United States, are culpably inattentive to the studies of their pupils; and that this is one of the causes which retard the improvement, and arrest the elevation of the profession. Exceptions to this (apparently invidious) remark, are frequently met with, especially in the great cities; but still they are *only* exceptions. Without presuming to inquire, at large, into the causes of this default, I may mention that which seems to me, the most operative. The price of tuition is too low. The preceptor feels no business obligation, and too often forgets what he owes to society and the profession. Like other men, he gives in proportion as he receives, and when a pupil comes without money, permits him to depart without knowledge. He would thus, by implication, cast the whole blame on the father, but the fault is equally his own. He ought not to take a student, without the design of making him a good practitioner, and when an inadequate fee is proffered by the father, should decline the application. If he does not, but graduates his aid according to the reward, he degrades the profession and is accessory to an imposture on the community. On this subject unfortunately we have no *esprit du corps*, no uniformity, no emulation; while the whole are imperiously required. The conscience which warms and purifies our professional efforts in the sick chamber, is too often dumb in the study, where its promptings are equally demanded. Tuition is an art, but not governed by fixed and universal laws. The student is to be urged onward from the 'known to the unknown' through a variety of complex and difficult sciences. In this proceeding, the mere *ipse dixit* of his preceptor can do but little good; while a well directed superintendance will confer lasting benefits. The kind and manner of assistance must necessarily vary with the idiosyncracies of both master and scholar; for that which facilitates the progress of one pupil, sometimes retards another, who requires a different regimen; and the methods which one preceptor follows with success, are often impracticable for another, from being inconsistent with his temperament and habits. But in the midst of these diversities of character, to which an excessive importance ought not to be attached, we may perceive, several methods which are applicable to every case, and I shall proceed to enumerate them.

1. The physician who proposes to become an instructor, should provide the requisite books, engravings, preparations and apparatus. He must not rely on his own attainments, as they cannot be transferred to his pupils, but through the instrumentality of established means.

2. He should form a plan of elementary studies and require his pupils to follow it. To this end he must designate every book that is to be read, and debar them from the perusal of any others. It is even necessary, to mark portions which should be passed over, as being incomprehensible without ampler preparation, or as having been proven erroneous. All our systematic works abound in chapters of the latter kind, the study of which cannot be too strongly reprobated, as planting errors in the young mind which may never be eradicated.

3. He should encourage his students to ask him questions and solicit his aid, on the obscure and difficult parts of every author; that nothing may be passed through without being understood.

4. As a further means of effecting the same object, he ought to examine every one on the book he has just finished; and, thus, not only correct his errors, but animate him to close and accurate application.

5. At least once a week he ought to assemble his pupils into a class, and subject them to systematic examinations on the studies in which they are engaged. When such examinations are ably conducted, they constitute the most interesting and valuable exercises in which students ever engage. By a series of well directed questions he may guide them through the labyrinths of the most complicated subject; and enable them to analyze that which they would otherwise find impenetrable and hopeless. Their errors of principle and nomenclature will be corrected; and their confidence in the truth established; their scattered acquirements solidified into a system, its deficiences made manifest, and the mode of supplying them indicated. Thus they will be led to contemplate and comprehend the relations which unite facts, that in badly educated minds remain insulated and in apparent variance. Above all, he can in this way and no other, relieve the *tedium* of solitary reading; invest the study with charms that render it attractive; and inspire an emulation for relative excellence, that will seldom fail to work out positive distinction.

6. He should require each pupil, at least once a month, to write a thesis. This exercise should commence with his studies, and continue till it terminates in his inaugural dissertation. Its advantages are manifold, not the least of which is the pleasure, which, in a short time, it affords to every aspiring and inquisitive student. When studying anatomy, his theses will of course be purely descriptive, a kind of writing in which none can be proficient without practice. As he advances, they may assume a higher and more diversified character. Some of them should be simple abridgements of chapters written with diffuseness, of which, unfortunately, there is no lack; others might be purely analytical; others critical, or in the manner of reviews; and a portion, mere commentaries on authors of a concise and aphoristick cast. In this way, he will form habits of attention, and be led to study with profit, what he would have passed by with carelessness. In the latter periods of study, his essays may take on a still higher character. He may begin to unite facts into regular dissertations, according to the rules of a sound philosophical logic; investigating principles, and constructing the elements of the system which is to guide and govern his future efforts in the practice.

Was the custom of writing enjoined on every pupil, we should not have so many candidates for graduation, incapable of composing their own theses; nor so many good physicians, who are unqualified to lay before the profession the results of their experience. Not a few of them, indeed, from neglecting what is here recommended, find themselves, in after life, unable to write a single sheet of professional directions with simplicity, taste and perspicuity.

7. Lastly. It is the duty of the preceptor, when his pupil is prepared for clinical observations, to select for him such cases as will be profitable. The Diagnosis of diseases presents many difficulties, and can be successfully studied only at the bed side. To introduce a student indiscriminately to all cases, is more likely to confuse than enlighten his mind. He should first study those which present a well defined character, and proceed gradually to the more obscure and complicated. But the most judicious selection of cases, will impart but little instruction, unless they are made the subjects of conversation and comment by the preceptor. In these clinical lectures, the benefit which he confers upon his disciple, if not greater, will be more deeply felt, than in any other period of their connexion. The utility of whathe has been teaching will now be made apparent; and the endearing relation finally dissolved, with feelings of gratitude in one, and self approving consciousness in the other.

